# Strategies targeting angiogenesis in advanced non-small cell lung cancer

**DOI:** 10.18632/oncotarget.17957

**Published:** 2017-05-17

**Authors:** Jun Wang, Jianpeng Chen, Yan Guo, Baocheng Wang, Huili Chu

**Affiliations:** ^1^ Department of Oncology, General Hospital, Jinan Command of the People’s Liberation Army, Jinan, China; ^2^ Department of Oncology, Shandong Provincial Hospital Affiliated to Shandong University, Jinan, China; ^3^ Department of Outpatient, Military Command of Shandong Province, Jinan, China

**Keywords:** NSCLC, angiogenesis, antiangiogenic agents, bevacizumab

## Abstract

Tumor angiogenesis is a frequent event in the development and progression of non-small cell lung cancer (NSCLC) and has been identified as a promising therapeutic target. The vascular endothelial growth factor (VEGF) family and other angiogenic factors, including fibroblast growth factor and platelet-derived growth factor, promote the growth of newly formed vessels from preexisting vessels and change the tumor microenvironment. To date, two antiangiogenic monoclonal antibodies, bevacizumab and ramucirumab, which target VEGF-A and its receptor VEGF receptor-2, respectively, have been approved for the treatment of locally advanced or metastatic NSCLC when added to first-line standard chemotherapy. Numerous oral multitargeting angiogenic small molecule tyrosine kinase inhibitors (TKIs) have been widely evaluated in advanced NSCLC, but only nintedanib in combination with platinum-based doublet chemotherapy has demonstrated a survival benefit in the second-line setting. Additionally, small-molecule TKIs remain the standard of care for patients with mutated EGFR, ALK or ROS1. Moreover, immune checkpoint inhibitors that target the programmed cell death protein 1 (PD-1) and programmed cell death protein ligand 1 (PD-L1) are changing the current strategy in the treatment of advanced NSCLC without driver gene mutations. The potential synergistic activity of antiangiogenic agents and TKIs or immunotherapy is an interesting topic of research. This review will summarize the novel antiangiogenic agents, antiangiogenic monotherapy, as well as potential combination therapeutic strategies for the clinical management of advanced NSCLC.

## INTRODUCTION

Non-small cell lung cancer (NSCLC) accounts for nearly 85% of all lung cancer cases and is one of the frequently diagnosed malignancies. It has a high mortality worldwide, and a majority of patients with NSCLC are initially diagnosed with stage III or IV disease and are no longer eligible for surgical resection. Moreover, for patients with advanced NSCLC, the 5-year survival rate is less than 5% [1]. Platinum-containing chemotherapy remains the standard care for advanced NSCLC that do not harbor mutations of driver genes in the first-line setting. However, most patients experience disease progression following the standard chemotherapy, and the benefits and efficacy of second-line treatment are limited [2]. Recently, broad studies of tumor biology have allowed developing particular targeted therapies for patients with specific mutations in multiple driver genes, including the epidermal growth factor receptor (EGFR), anaplastic lymphoma kinase (ALK), as well as ROS proto-oncogene 1 (ROS1). In the past 10 years, several randomized trials have established tyrosine kinase inhibitors (TKIs), such as gefitinib, erlotinib, afatinib and crizotinib, as approved first-line drugs in NSCLC with targetable driver mutations or rearrangements [3–6]. Moreover, approximately 20-30% of patients with NSCLC display primary resistance to these target inhibitors and lack an excellent initial clinical activity, although they have a sensitive gene mutation. In addition, patients with sensitive mutations ultimately develop secondary resistance to these drugs after several months of therapy [7]. Thus, the need for new therapeutic strategies for advanced NSCLC is urgent.

Angiogenesis refers to the growth of newly formed blood vessels from the pre-existing vasculature. This complex physiological process involves a dynamic balance between angiogenesis inducers and inhibitors that tightly coordinate with macrophages, endothelial cells, and pericytes. However, the tumor tends to change this balance towards releasing chemical signals that stimulate angiogenesis and induce other cells to produce high levels of pro-angiogenesis factors in the tumor microenvironment, which promote cancer invasion and metastasis. Tumor angiogenesis results in abnormally formed, tortuous, and poorly organized vessels that exhibit altered permeability [8, 9]. Specifically, microenvironment hypoxia within the tumor induces the expression of multiple angiogenesis-related molecules, including the vascular endothelial growth factor (VEGF), platelet-derived growth factor (PDGF), and fibroblast growth factor (FGF) families [10–12]. Of these molecules, VEGF-A represents a dominant angiogenesis promoter that stimulates the proliferation of endothelial cell, migration, and the formation of new blood vessels, mainly by interacting with its receptor VEGF receptor-2 (VEGFR-2) [13].

In fact, increased micro-vessel density and elevated circulating expression of VEGF-A are significantly related to a poor survival in lung cancer [14, 15]. Therefore, the angiogenesis pathway has been viewed as an important therapeutic target in lung cancer and other cancer types [16]. The tumor mass of nutrients to tumor growth is significantly deprived *via* booking angiogenesis, with a normalization of newly formatted vessels. At present, antiangiogenic treatment can be based on two major strategies: blocking the pro-angiogenesis pathway and enhancing the levels of antiangiogenic factors [17]. Monoclonal antibodies that block the function of VEGF-A or its receptor VEGFR-2 and different small-molecule multitargeting TKIs that block VEGFR and other receptor-mediated signaling pathways have been discovered and developed in clinical practice. For example, bevacizumab is a humanized monoclonal antibody targeting VEGF, has been approved by the US Food and Drug Administration (FDA) as a standard regimen for advanced NSCLC in the first-line setting. The FDA has also approved an antibody targeting VEGFR-2, ramucirumab, plus docetaxel for metastatic NSCLC that has progressed after first-line therapy. Endostar, a recombinant human endostatin, has been approved by the China FDA in 2005 for the therapy of metastatic NSCLC. It specifically promotes cell apoptosis and potently inhibits endothelial cell proliferation and tumor growth. In this review, we will summarize the current state and recent advances in the clinical treatment of advanced NSCLC with angiogenesis inhibitors, including the combination of antiangiogenic therapy and chemotherapy (Table 1 and 2), the combination of antiangiogenic therapy and EGFR TKIs (Table 3) or immune checkpoint inhibitors (Table 4), and the use of antiangiogenic agents alone (Table 5).

**Table 1:** Trials evaluating bevacizumab or ramucirumab in combination with chemotherapy in locally advanced or metastatic NSCLC

Study	Design	Patients	n	Study arm	Control arm	mPFS/mTTP	mOS	ORR	PE, *P* value
First-line									
Johnson et al. [18]	Phase II	NSCLC	99	Pac+Car+Bev	Pac+Car	7.4 (7.5 mg/kg) vs 4.3 (15 mg/kg) vs 4.2 m	17.7 (7.5 mg/kg) vs 11.6 (15 mg/kg) vs 14.9 m	31.5% (7.5 mg/kg) vs 28.1% (15 mg/kg) vs 18.8%	TTP; *p* = 0.023 (15 mg/kg)
ECOG 4599 [19]	Phase III	nsNSCLC	878	Pac+Car+Bev	Pac+Car	6.2 vs 4.5 m	12.3 vs 10.3 m	35% vs 15%	OS; *p* = 0.003
AVAIL [20, 21]	Phase III	nsNSCLC	1,043	Gem+Cis+Bev	Gem+Cis	6.7 (7.5 mg/kg) vs 6.5 (15 mg/kg) vs 6.1 m	13.6 (7.5 mg/kg) vs 13.4 (15 mg/kg) vs 13.1 m	34.1% (7.5 mg/kg) vs 30.5% (15 mg/kg) vs 20.1%	PFS; *p* = 0.0003 (7.5 mg/kg), P = 0.0154 (15 mg/kg)
BEYOND [23]	Phase III	nsNSCLC	276	Pac+Car+Bev	Pac+Car	9.2 vs 6.5 m	24.3 vs 17.7 m	54.4 vs 23.3%	OS; *p* = 0.0154
JO19907 [22]	Phase II	nsNSCLC	180	Pac+Car+Bev	Pac+Car	6.9 vs 5.9 m	22.8 vs 23.4 m	60.7% vs 31%	PFS; *p* = 0.009
SAiL [24–26]	Phase IV	nsNSCLC	2,212	Patinum-based chemotherapy+Bev		7.8 m	14.6 m	51%	
Camidge et al. [41]	Phase II	NSCLC	22	Pal+Car+Ram		7.85 m	16.85 m	55%	6-month PFS: 59%
Doebele et al. [42]	Phase II	nsNSCLC	140	Pem+Pla+Ram	Pem+Pla	7.2 vs 5.6 m	13.9 vs 10.4 m	49.3% vs 38.0%	PFS; *p* = 0.132
Maintenance									
Leon et al. [30]	Phase II	nsNSCLC	49	Vin+Cis+Bev→Bev		6 m	14.7 m	29%	PFS
Stevenson et al. [31]	Phase II	nsNSCLC	43	Pem+Car+Bev→Bev		7.1 m	17.1 m	47%	PFS
Patel et al. [32]	Phase II	nsNSCLC	50	Pem+Car+Bev→Pem+Bev		7.8 m	14.1 m	55%	PFS
AVAPERL [33, 34]	Phase III	nsNSCLC	376	Pem+cis+Bev→Pem+Bev	Pem+cis+Bev→Bev	7.4 vs 3.7 m	17.1 vs 13.2 m	55.5% vs 50.0%	PFS; *p* < 0.0001
POINTBREAK [35]	Phase III	nsNSCLC	939	Pem+Car+Bev→Pem+Bev	Pac+Car+Bev→Bev	6.0 vs 5.6 m	13.4 vs 12.6 m	34.1% vs 33.0%	OS; *p* = 0.949
PRONOUNCE [36]	Phase III	nsNSCLC	371	Pac+Car+Bev→Bev	Pem+Car→Pem	3.91 vs 2.86 m	11.7 vs 10.5 m	23.6% vs 27.4%	G4PFS, *p* = 0.176
Second-line									
Herbst et al. [37]	Phase II	nsNSCLC	81	Doc/Pem+Bev	Doc/Pem+Bev+Plac	4.8 vs 3.0 m	12.6 vs 8.6 m	12.5% vs 12.2%	PFS; HR: 0.38 (95%CI: 0.38-1.16)
REVEL [43]	Phase III	NSCLC	1,253	Doc+Ram	Doc+Plac	4.5 vs 3.0 m	10.5 vs 9.1 m	23% vs 14%	OS; *p* = 0.023
Yoh [44]	Phase II	NSCLC	197	Doc+Ram	Doc+Plac	5.22 vs 4.21 m	15.15 vs 14.65 m	28.9% vs 18.5%	PFS; 0.83 (0.59-1.16)

NSCLC: non-small cell lung cancer; nsNSCLC: non-squamous non-small cell lung cancer; mPFS: median progression-free survival; mTTP: median time to progression; ORR: objective response rate; PE: Primary endpoint; Pac: paclitaxel; Car: carboplatin; Bev: bevacizumab; Ram: ramucirumab; Gem: Gemcitabine; Cis: cisplatin; Pla: platinum; Doc: docetaxel; Plac: placebo; G4PFS: PFS without grade 4 toxicity; HR: hazard ratio

**Table 2:** Trials evaluating antiangiogenic TKIs in combination with chemotherapy in locally advanced or metastatic NSCLC as first or second-line therapy

Study	Design	Patients	*n*	Experimental arm	Control arm	mPFS/mTTP	mOS	ORR	PE, *p* value
First-line									
ESCAPE [49]	Phase III	NSCLC	926	Pac+Car+Sor	Pac+Car	4.6 vs 5.4 m	10.7 vs 10.6 m	27.4% vs 24.0%	OS; *p* = 0.915
NEXUS [50]	Phase III	nsNSCLC	772	Gem+Cis+Sor	Gem+Cis	6.0 vs 5.5 m	12.4 vs 12.5 m	28% vs 26%	OS; *p* = 0.401
MONET1 [51]	Phase III	nsNSCLC	1090	Pac+Car+Mot	Pac+Car	5.6 vs 5.4 m	13.0 vs 11.0 m	40% vs 26%	OS; *p* = 0.14
NCT00369070 [52]	Phase II	nsNSCLC	186	Pac+Car+Mot	Pac+Car+Bev	7.7 (125 mg qd) vs 5.8 (75 mg bid) vs 8.3 m	14.0 (125 mg qd) vs 12.8 (75 mg bid) vs 14.0	30% vs 23% vs 37%	ORR
NCIC IND [53]	Phase I	NSCLC	20	Pac+Car+Ced		7.6 m		45%	
BR24 [54]	Phase II	NSCLC	251	Pac+Car+Ced	Pac+Car	5.6 vs 5.0 m			PFS; *p* = 0.08
BR29 [55]	Phase III	NSCLC	306	Pac+Ced	Pac	5.5nvs 5.5 m	12.2 vs 12.1 m	52% vs 34%	OS; *p* = 0.72
N0528 [56]	Phase II	NSCLC	87	Gem+Cb+Ced	Gem+Car	6.3 vs 4.5 m	12 vs 9.9 m	19% vs 20%	ORR; *p* = 1.0
Heymach [57]	Phase II	NSCLC	108	Pac+Cb+Van	Pac+Car	24 vs 23 w	10.2 vs 12.6 m	32% vs 25%	PFS; *p* = 0.098
Aisner et al. [58]	Phase II	NSCLC	162	Pac+Cb+Van→van	Pac+Car+Van→Plac	4.5 vs 4.2 m	9.8 vs 9.4 m		PFS; *p* = 0.07
Scagliotti et al. [59]	Phase II	nsNSCLC	106	Pem+Paz	Pem+Cis	25.0 vs 22.9 w	HR: 1.22; P = 0.55	23% vs 34%	PFS; *p* = 0.26
Belani et al. [60]	Phase II	nsNSCLC	170	Pem+Cis+Axi	Pem+Cis+Axi	8.0 (d1-21) vs 7.9 (d2-19) vs 7.1 m	16.6 (d1-21) vs 14.7 (d2-19) vs 15.9 m	45.5% (d1-21) vs 39.7% (d12-19) vs 26.3%	PFS; *p* = 0.36 (d1-21); p = 0.54 (d2-19)
Twelves et al. [61]	Phase II	nsNSCLC	118	Pac+Car+Axi	Pac+Car+Bev	5.7 vs 6.1 m	10.6 vs 13.3 m	29.3% vs 43.3%	PFS; *p* = 0.64
Ramalingam et al. [62]	Phase II	nsNSCLC	138	Pac+Car+Lin	Pac+Car	8.3 (7.5 mg) vs 7.3 (12.5 mg) vs 5.4 m	11.4 (7.5mg) vs 13.0 (12.5 mg) vs 11.3 m	8.3 (7.5 mg) vs 7.3 (12.5 mg) vs 5.4 m	PFS; *p* = 0.022 (7.5 mg); *p* = 0.118 (12.5 mg)
Second-line									
N0626 [63]	Phase II	NSCLC	100	Sor+Pem	Pem	3.4 vs 4.1m	9.4 vs 9.1m		PFS; *p* = 0.22
CALGB30704 [64]	Phase II	NSCLC	130	Pem+Sun	Pem; Sun	3.7 vs 4.9 vs 3.3 m (Sun alone)	6.7 vs 10.5 vs 8.0 m (Sun alone)	22% vs 17% vs 14 (Sun alone)	PFS; *p* = 0.25
LUME-lung 1 [65]	Phase III	NSCLC	1,311	Doc+Nin	Doc	3.4 vs 2.7 m	10.0 vs 9.1 m	4.4% vs 3.3%	PFS; *p* = 0.0019
LUME-lung 2 [66]	Phase III	nsNSCLC	713	Pac+Nin	Pac	4.4 vs 3.6 m	12.0 vs 12.7 m	9.1% vs 8.3%	PFS; *p* = 0.0435
ZODIAC [65]	Phase III	NSCLC	1,391	Doc+Van	Doc	4.0 vs 3.2 m	10.6 vs 10.0 m	17% vs 10%	PFS; *p* < 0.0001
ZEAL [66]	Phase III	nsNSCLC	534	Pem+Van	Pem	17.6 vs 11.9 w	10.5 vs 9.2 m	19% vs 8%	PFS; *p* = 0.108

NSCLC: non-small cell lung cancer; nsNSCLC: non-squamous non-small cell lung cancer; mPFS: median progression-free survival; mTTP: median time to progression; ORR: objective response rate; DCR: disease control rate; PE: Primary endpoint; Pac: paclitaxel; Car: carboplatin; Bev: bevacizumab; Ram: ramucirumab; Cis: cisplatin; Pla: platinum; Doc: docetaxel; Plac: placebo; Sor: Sorafenib; Mot; Motesanibb; Ced: cediranib; Van: Vandetanib; Paz: pazopanib; Axi: axitinib;; Lin: Linifanib; Sun: sunitinib; Nin: nintedanib; Erl: erlotinib; Lin: linifanib

**Table 3:** Trials evaluating antiangiogenic agents in combination with EGFR TKIs in advanced NSCLC

Study	Design	Patients	*n*	Study arm	Control arm	mPFS/mTTP	mOS	ORR	PE (*P*)
First-line									
Ichihara et al. [78]	Phase II	NSCLC	42	Gef+Bev		14.4 m	Immature	73.8%	1-year PFS: 56.7%
JO25567 [76]	Phase II	NSCLC	154	Erl+Bev	Erl	16.0 vs 9.7 m	Immature	69% vs 64%	PFS; *p* = 0.0015
BELIEVE [77]	Phase II	NSCLC	109	Erl+Bev		13.8 m	Immature	76.1%	PFS
RELAY[83]	Phase Ib/III	Ongoing							
Maintenance									
ATLAS [79]	Phase III	NSCLC	1,155	Chemo+Bev→Bev+Erl	Chemo+Bev→Bev+Plac	4.8 vs 3.7 m	14.4 vs 13.3 m		PFS; *p* < 0.001
Second-line									
Herbst et al. [37]	Phase II	nsNSCLC	81	Erl+Bev	Doc/Pem	4.4 m vs 3.0 m	13.7 vs 8.6m	17.9% vs 12.2%	PFS; HR: 0.72; 95%CI: 0.42-1.23
Beta [80]	Phse III	NSCLC	636	Erl+Bev	Erl+Plac	3.4 vs 1.7m	9.3 vs 9.2 m	13% vs 6%	OS; *p* = 0.758
Groen et al. [85]	Phase II	NSCLC	132	Sun+Erl	Sun+Plac	2.8 vs 2.0 m	8.2 vs 7.6 m	4.6% vs 3.0%	PFS; *p* = 0.321
Scagliotti et al. [86]	Phase III	NSCLC	960	Sun+Erl	Sun+Plac	3.6 vs 2.0 m	9.0 vs 8.5 m	10.6% vs 6.9%	OS; *p* = 0.1388
Spigel et al. [84]	Phase II	NSCLC	168	Sor+Erl	Erl+Plac	3.38 vs 1.94 m	7.62 vs 7.23 m	8% vs 11%	ORR (*p* = 0.56); PFS (*p* = 0.196)

NSCLC: non-small cell lung cancer; nsNSCLC: non-squamous non-small cell lung cancer; mPFS: median progression-free survival; mTTP: median time to progression; ORR: objective response rate; PE: Primary endpoint; Gef: gefitinib; Bev: bevacizumab; Erl: erlotinib; Sun: sunitinib; Plac: placebo; Doc: docetaxel; Pem: pemetrexed

**Table 4:** Trials evaluating antiangiogenic agents in combination with immune checkpoint inhibitors in locally advanced or metastatic NSCLC

Study	Design	Patients	Estimated enrollment (total)	Interventions	Primary endpoints	Start date	Estimated completion date
NCT02039674	Phase I/II	Untreated unresectable or metastatic NSCLC	308	Pembrolizumab plus bevacizumab and/or chemotherapy (paclitaxel and carboplatin)	Safety, tolerability, and efficacy	February 2014	June 2019
NCT02681549	Phase II	Metastatic melanoma or NSCLC with untreated brain metastases, and with any number of previous systematic treatments with the exception of previous inhibitors of PD-1, PD-L1, or PD-L2.	53	Bevacizumab plus pembrolizumab	Brain metastasis response rate	May 2016	May 2019
NCT02366143	Phase III	Untreated stage IV non-squamous NSCLC	1,200	Atezolizumab plus bevacizumab plus paclitaxel plus carboplatin	Progression-free survival	March 2015	November 2022
NCT01454102 (CheckMate 012)	Phase I	Untreated advanced NSCLC	412	Nivolumab plus bevacizumab as maintenance therapy	Safety and efficacy	December 2011	November 2017
NCT01633970	Phase Ib	Locally advanced or metastatic solid tumors including NSCLC	225	Atezolizumab plus bevacizumab and/or with chemotherapy (FOLFOX)	MDT of Atezolizumab/AEs/ DLTs	July 2012	December 2018
NCT02443324	Phase Ia/b	Patients with gastric or GEJ adenocarcinoma, NSCLC or transitional cell carcinoma of the urothelium	155	Ramucirumab plus pembrolizumab	DLTs	July 2015	December 2017
NCT02856425	Phase Ib	Advanced NSCLC progressed on at least one prior line of chemotherapy	18	Nintedanib plus pembrolizumab	MDT	July 2016	July 2021

NSCLC: non-small cell lung cancer; PD-1; programmed cell death protein 1; PD-L1/2: programmed cell death protein ligand 1/2; MDT: maximum tolerated dose; GEJ, gastroesophageal junction; DLTs: dose limiting toxicities; AEs, adverse events; F, fluorouracil (5-FU); FOL, folinic acid (leucovorin); OX, oxaliplatin

**Table 5:** Trials evaluating antiangiogenic agent alone in locally advanced or metastastic NSCLC as first or second-line therapy

Study	Design	Patients	*n*	Experimental arm	Control arm	mPFS/mTTP	mOS	ORR	PE, *p* value
MISSIN [107]	Phase II	NSCLC	703	Sor	Plac	2.8 vs 1.4 m	8.2 vs 8.3 m	4.9% vs 0.9%	PFS; *p* = 0.47
CTONG 0805 [109]	Phase II	NSCLC	65	Sor		3.7 m	7.4 m	3.1%	ORR: 32.8%
NCT00922584 [110]	Phase II	NSCLC	52	Sor		2.7 m	6.7 m	0%	ORR
E2501 [111]	Phase II	NSCLC	105	Sor	Plac	3.3 vs 2.0 m	13.7 vs 9.0 m	2% vs 3%; DCR (54% vs 23%)	DCR; *p* = 0.005
ZEST [112]	Phase III	NSCLC	1,240	Van	Erl	2.6 vs 2.0 m	6.9 vs 7.8 m	12% vs 12%	PFS; *p* = 0.721
ZEPHYR [108]	Phase III	NSCLC	924	Van	Plac	1.9 vs 1.8 m	8.5 vs 7.8 m	2.6% vs 0.7%	OS; *p* = 0.527
Reck et al. [113]	Phase II	NSCLC	73	Nin 150 mg bid or Nin 250 mg bid		53 (150 mg bid); 48d (250 mg bid)	20.6 (150 mg bid); 20.7w (250 mg bid)	0% (150 mg bid); 2.8% (250 mg bid)	PFS (6.9 w) and ORR (1.4%)
Tan et al. [114]	Phase II	NSCLC	139	Lin		3.6 m	9.0 m	5.0%	PFS at 16 weeks (33.1%)

NSCLC: non-small cell lung cancer; mPFS: median progression-free survival; mTTP: median time to progression; OS: overall survival; ORR: objective response rate; DCR: disease control rate; PE: Primary endpoint; Plac: placebo; Sor: Sorafenib; Van: Vandetanib; Lin: Linifanib; Nin: nintedanib; Erl: erlotinib; Lin: linifanib

## COMBINATION OF ANTIANGIOGENIC AGENTS AND CHEMOTHERAPY

### Bevacizumab plus chemotherapy

As a humanized monoclonal antibody targeting VEGF-A, bevacizumab suppresses the binding of VEGF-A to its receptors to prevent its proangiogenic activity. The results from an early-randomized phase II trial showed that adding bevacizumab to standard doublet chemotherapy produced a higher objective response rate (ORR) and longer median time to progression (TTP) [18]. However, the median overall survival (OS) was similar between the bevacizumab and standard chemotherapy group. Moreover, hemoptysis events were observed in bevacizumab group, especially in a subset of patients with large tumors adjacent to major vessels or cavitary tumors, and patients with squamous cell histology. The subsequent ECOG 4599 study was the first randomized phase III trial evaluating first-line paclitaxel and carboplatin chemotherapy plus bevacizumab (*n* = 439) *versus* chemotherapy alone (*n* = 439) for NSCLC patients [19]. In particular, only cases with nonsquamous histology were enrolled in this trial. This study showed a significantly improved OS of 12.3 months in the combination arm *versus* 10.3 months in the chemotherapy arm. The ORR (35% *versus* 15%) and PFS significantly differed between the two arms. The combination regimen was well tolerated, but more grade 3 or 4 bleeding events occurred in 4.4% of patients with bevacizumab. Other main adverse events in the combination arm included neutropenia, hypertension, febrile neutropenia and proteinuria. In the AVAiL phase III trial, bevacizumab was also evaluated as an addition to gemcitabine and cisplatin chemotherapy in metastatic nonsquamous patients [20, 21]. A total of 1043 patients were enrolled to receive chemotherapy plus 7.5 mg/kg or 15 mg/kg bevacizumab or chemotherapy alone. Patients receiving bevacizumab and chemotherapy experienced prolonged PFS. However, prolonged OS was not observed in the 7.5 mg/kg or 15 mg/kg bevacizumab group compared to the group receiving gemcitabine and cisplatin chemotherapy alone. The phase II JO19907 trail evaluated the efficacy of paclitaxel-carboplatin plus bevacizumab or placebo in Japanese patients with metastatic nonsquamous NSCLC. The ORR was 61% for bevacizumab compared with 31% for bevacizumab plus chemotherapy [22]. In the phase III BEYOND trail enrolling 276 Chinese patients, PFS was significantly different between the combination group (gemcitabine-cisplatin plus bevacizumab) and the group receiving chemotherapy alone (9.2 *versus* 6.5 months), and the ORR (54% *versus* 26%) and OS (24.3 *versus* 17.7 months) were also significantly different between the two study arms [23]. The large SAiL study enrolling 2,212 patients confirmed that the combination therapy with bevacizumab and platinum-based chemotherapy has a manageable safety profile and offered a clinical survival benefit to patients with advanced NSCLC [24]. Subsequent subgroup analyses revealed that the safety and efficacy in Asian or Chinese populations were consistent with those observed in several previous phase III trials [25, 26]. Recently, two meta-analyses proved that bevacizumab addition prolongs OS when it was added to doublet platinum-containing chemotherapy in first-line setting [27, 28]. The addition of bevacizumab decreased the risk of mortality by nearly10%. In 2006, bevacizumab received FDA approval for treating patients with stage IV NSCLC. Subsequently, this antibody was also approved by the European Medicine Agency (EMA) for advanced nonsquamous NSCLC in the first-line setting. Additionally, retrospective data from the ECOG 4599 and the US Oncology network show that the continual use of bevacizumab until disease progression prolonged both PFS and OS [19, 29].

Many phase II trials have also evaluated the efficacy of bevacizumab maintenance for metastatic NSCLC after induction treatment with bevacizumab plus different platinum-based chemotherapy regimens, such as bevacizumab alone [30, 31] or combination with pemetrexed [32]. In several phase III trials, bevacizumab was also studied as a maintenance therapy after its association with induction chemotherapy with platinum-based regimen. In the AVAPERL randomized phase III trial, NSCLC patients receiving bevacizumab and pemetrexed maintenance therapy had a longer PFS than patients receiving bevacizumab maintenance (7.4 *versus* 3.7 months), but OS only numerically differed between the two groups (17.1 *versus* 13.2 months) [33, 34]. The POINTBREAK study evaluated the efficacy and safety of pemetrexed plus carboplatin plus bevacizumab followed by maintenance treatment with pemetrexed and bevacizumab *versus* those of paclitaxel plus carboplatin plus bevacizumab followed by maintenance treatment with bevacizumab in advanced nonsquamous NSCLC [35]. This trial failed to reach its primary endpoint of OS, although improvements were observed in PFS and the ORR. In the PRONOUNCE trial, after chemotherapy with pemetrexed and carboplatin, maintenance treatment with pemetrexed did not prolong PFS without grade 4 toxicity compared to maintenance treatment with bevacizumab after paclitaxel plus carboplatin plus bevacizumab [36]. Overall, these trials fail to present sufficient data to identify the optimal regimen in the maintenance treatment setting (cytotoxic chemotherapy, bevacizumab, or cytotoxic chemotherapy plus bevacizumab). A randomized phase III ECOG 5508 trial would help to identify the superior treatment, the combination of pemetrexed and bevacizumab *versus* pemetrexed or bevacizumab monotherapy, as a switch maintenance approach beyond standard chemotherapy.

Second-line treatment strategies are currently limited and include docetaxel or pemetrexed chemotherapy alone, with response rates of < 10%. Investigators have also evaluated the second-line combination of chemotherapy with bevacizumab *versus* chemotherapy alone. A phase II trial showed that the risk of disease progression or mortality was decreased by 34% in the chemotherapy plus bevacizumab group compared to patients treated with the chemotherapy group. Moreover, the one-year survival rate was 53.8% in the bevacizumab plus chemotherapy group *versus* 33.1% in the chemotherapy group [37]. The AVaALL trial is ongoing and assessing the efficacy of continuation of bevacizumab after disease progression in advanced NSCLC receiving 4-6 cycles of standard therapy with bevacizumab and chemotherapy in the first-line setting. The OS is the primary endpoint (NCT01351415) [38]. Overall, the confirmed role of bevacizumab in the second-line treatment for advanced NSCLC remains unclear.

### Ramucirumab plus chemotherapy

Ramucirumab is a recombinant human monoclonal antibody that inhibits angiogenesis by targeting the VEGFR-2 signaling pathway. It is different from bevacizumab, which specifically targets the VEGFR-2 ligand VEGF. The FDA approved its use for metastatic gastric or gastro-esophageal junction carcinoma with paclitaxal in the second-line treatment based on data from the RAINBOW published in 2014 [39] and for metastatic colorectal cancer with FOLFIRI based on data from the RAISE trial published in 2015 [40].

The combination of ramucirumab and standard platinum-containing chemotherapy was also evaluated in metastatic NSCLC as a first-line treatment. Camidge et al. conducted a first phase II trial that evaluated the efficacy of ramucirumab when combined with chemotherapy with paclitaxel and carboplatin in advanced NSCLC [41]. Specifically, a total of 40 patients were enrolled and received 10 mg/kg ramucirumab followed by 200 mg/m^2^ paclitaxel and carboplatin. The estimated median PFS and OS was 7.85 and 16.9 months, respectively. The efficacy of ramucirumab was consistent with that reported by the ECOG 4599 study, in which bevacizumab plus chemotherapy improved the median PFS from 4.5 to 6.2 months. Another randomized phase II trial investigated whether the addition of ramucirumab to pemetrexed plus platinum chemotherapy increase the efficacy in advanced nonsquamous NSCLC [42]. PFS was designed as the primary endpoint. A total of 140 patients were enrolled to receive treatment with pemetrexed plus platinum (cisplatin or carboplatin) or pemetrexed and platinum plus ramucirumab. Unfortunately, this study did not reach its primary endpoint of significant prolongation of PFS; the median PFS in the chemotherapy arm was 5.6 months and 7.2 months in the ramucirumab plus chemotherapy arm. The ORR was similar between the ramucirumab plus chemotherapy and chemotherapy alone groups (49.3% *versus* 38.0%), but the addition of ramucirumab to chemotherapy increased the disease control rate.

Subsequently, the REVEL trial evaluated the effect of ramucirumab plus chemotherapy on metastatic NSCLC as a second-line therapy [43]. A total of 1,253 NSCLC patients who progressed after first-line platinum-based chemotherapy received docetaxel alone or docetaxel plus ramucirumab. This study did not exclude a group of patients who had received first-line bevacizumab or those with squamous histology. Fortunately, this study reached its primary endpoint, with an improved median OS of 10.5 months for combination treatment compared to 9.1 months for docetaxel chemotherapy. The median PFS was 4.5 in the combination arm and 3.0 months in the docetaxel arm, respectively. The ORR also differed between the two groups (23% *versus* 14%). Moreover, survival benefits were observed for a subgroup of patients with squamous or nonsquamous histology. Furthermore, a randomized phase II study in Japanese NSCLC patients who progressed on first-line chemotherapy demonstrated that the median PFS was longer in the ramucirumab plus docetaxel group (5.2 months; *n* = 76) than that in the placebo plus docetaxel group (4.2 months; *n* = 81), although the median OS (15.5 months with ramucirumab plus docetaxel; 14.7 months with placebo plus docetaxel) and ORR were similar in the two groups. Thus, the data from this Japanese trial were similar to those obtained from the REVEL trial and demonstrated a manageable safety profile [44]. Based on the data from the REVEL trial, the combination of ramucirumab and docetaxel was approved by the FDA as a treatment strategy for metastatic NSCLC in the second-line setting that has progressed after first-line therapy.

### Vascular disrupting agent plus chemotherapy

Unlike bevacizumab and ramucirumab that reduce tumor vessel density and induce maturation of vessels during antiangiogenic therapy by ‘vascular normalization’, vascular disrupting agents specifically target preexisting vasculature through selective occlusion of tumor vessels or ligand-directed disrupting with toxins or pro-coagulant agents. Lara et al. evaluated the efficacy of novel vascular disrupting agent ASA404 (vadimezan) with or without first-line chemotherapy in NSCLC patients. Although the addition of ASA404 to first-line chemotherapy with carboplatin and paclitaxel generally well tolerated, but OS was similar in two arms [45]. Another vascular disrupting agent aflibercept was a recombinant human fusion protein targeting the VEGF pathway, did not improve OS (10.1 months for aflibercept and 10.4 for placebo; *p* = 0.9) when it was added to second-line docetaxel chemotherapy in advanced or metastatic NSCLC, although PFS was different between the combination group (5.2 months) and chemotherapy group (4.1 months; *p* = 0.0035) [46].

### VEGFR-TKI plus chemotherapy

Small-molecule antiangiogenic agents plus chemotherapy has become another combination therapeutic strategy for advanced NSCLC. Unfortunately, in the first-line setting these drugs have failed to improve the therapeutic potential of standard chemotherapy (Table 2). Additionally, increased toxicity and fatal events, which are associated with antiangiogenic TKIs, limit the use of full doses when combined with cytotoxic chemotherapy.

Sorafenib was the first antiangiogenic TKI to be studied in lung cancer. As a multitargeting inhibitor of angiogenesis that targets VEGFR-2, Raf, PDGFR, and kit, sorafenib has been approved as a treatment choice for advanced hepatocellular carcinoma [47, 48]. Two phase III randomized studies also evaluated the efficacy of sorafenib plus standard chemotherapy in previously untreated metastatic NSCLC. In the ESCAPE trial, patients were enrolled to receive chemotherapy plus sorafenib or placebo [49]. Unfortunately, this trial was stopped because an interim analysis demonstrated no improvement in OS. The final data showed the median OS was similar between the chemotherapy plus sorafenib arm and chemotherapy plus placebo arm for all NSCLC histologic types (10.7 *versus* 10.6 months). In addition, the ORR and PFS were also similar between the two arms, and a planned analysis showed that in a subgroup of patients with squamous histology, chemotherapy plus sorafenib produced a worse OS (8.9 *versus* 13.6 months) and PFS than chemotherapy and placebo. Specifically, this subgroup of patients exhibited higher rates of thrombocytopenia, hand-food reaction, hypertension and pruritus when receiving sorafenib compared with placebo. The subsequent NEXUS trial, which compared sorafenib plus gemcitabine and cisplatin in previously untreated advanced NSCLC, did not include cases with squamous histology based on the results of the ESCAPE trial [50]. In the NEXUS trial, sorafenib did not improve the median OS (12.4 *versus* 12.8 months), and the toxicity profile were consistent with that found in the ESCAPE trial. Moreover, other antiangiogenic TKIs in combination with chemotherapy have failed to produce a meaningful survival benefit and were associated with increased cumulative toxicity profiles in the first-line or second-line setting, including vandetanib, cediranib, sunitinib, motesanib, pazopanib, linifanib and axitinib [51–66]. In addition, treatment with these antiangiogenic TKIs caused a higher incidence of toxicity.

However, nintedanib is an exception to these negative findings. Only nintedanib in combination with docetaxel showed a significant survival benefit. Nintedanib is a multitargeting antiangiogenic TKI that blocks the VEGF, PDGF and FGF signaling pathways. The randomized LUME-Lung 1 trial evaluated nintedanib plus docetaxel *versus* docetaxel alone as a second-line therapy for 1,314 metastatic NSCLC patients [67]. The primary and secondary endpoints were PFS and OS, respectively. The results showed that the combination of nintedanib plus docetaxel improved survival after the failure of the first-line chemotherapy. Specifically, patients in the nintedanib and docetaxel arm exhibited improved PFS compared to patients in the docetaxel arm. Moreover, nintedanib plus docetaxel significantly increased the median OS in a pre-specified subset of patients with adenocarcinoma (12.6 *versus* 10.3 months), and the combination treatment improved the OS in patients with adenocarcinoma who developed progressive disease within 9 months following the start of front-line therapy in a pre-specified subgroup analysis. The LUME-Lung 2 trial compared the combination of nintedanib with pemetrexed to placebo-pemetrexed in the treatment of advanced nonsquamous NSCLC following the failure of one prior line of treatment with chemotherapy [68]. However, enrollment was halted prematurely because of a lack of improvement in the investigator-assessed PFS. The independent centrally reviewed PFS was 4.4 months for nintedanib-pemetrexed *versus* 3.6 months for placebo-pemetrexed. The adverse event profile associated with nintedanib and docetaxel treatment was expected from these trials. Grade 3 or worse adverse events, including elevated alanine aminotransferase and aspartate aminotransferase levels and diarrhea, were manageable or reversible. The phase III LUME Columbus study (NCT02231164) was designed to compare the combination of nintedanib with docetaxel to docetaxel alone in NSCLC of adenocarcinoma histology after first-line chemotherapy. However, this study has been terminated because of an increased incidence of grade 5 toxicities (16.4% *versus* 11.8%).

### Rh-endostatin plus chemotherapy

The antiangiogenic agent rh-endostatin (Endostar) is more stable and potent than endocrine endostatin because of the addition of nine amino acids to the N terminus of endocrine endostatin [69]. Rh-endostatin has been found to suppress the migration of vascular endothelial cells and induce cell apoptosis. The results from a randomized, phase III trial conducted in China demonstrated a significant improvement in TTP for untreated advanced NSCLC with rh-endostatin plus vinorelbine and cisplatin chemotherapy. Significant improvements were also observed in ORR, the clinical benefit rate and quality of life score in the group treated with rh-endostain in combination with chemotherapy [70]. A phase II trial showed that the addition of rh-endostain to paclitaxel-carboplatin chemotherapy improved the ORR (39.3% *versus* 23.0%) and the disease control rate (90.2% *versus* 67.2%), but neither PFS nor OS significantly differed between the two arms [71]. Moreover, a meta-analysis of platinum-based chemotherapy with or without rh-endostain demonstrated significant improvements in the ORR and TTP, with manageable toxicity profiles [72]. Based on these data, the China Food and Drug Administration approved rh-endostain combined with chemotherapy as a first-line treatment for advanced NSCLC.

## COMBINATION OF ANTIANGIOGENIC AGENTS AND EGFR TKIs

### Bevacizumab plus EGFR TKIs

First and second-generation EGFR TKIs, including erlotinib, gefitinib and afatinib, have been shown to prolong PFS, increase clinical response, and relieve clinical symptoms compared with standard chemotherapy for patients with advanced NSCLC expressing mutant EGFR in the first-line setting [3–5]. Thus, the ability of the dual inhibition of both the VEGF and EGFR pathways to improve outcomes in a subgroup of patients expressing mutant EGFR warrants research [73]. In fact, preclinical data supported that EGFR is also expressed in the endothelial cells of tumor vessels and associated with tumor-induced VEGF expression and neovasculature [74]. Moreover, anti-VEGF treatment inhibited EGFR autocrine signaling, suggesting that the dual inhibition of EGFR and VEGF may lead to an increasing or synergistic activity [75].

A phase II trial (JO25567) conducted in Japan evaluated the efficacy and safety of first-line erlotinib plus bevacizumab *versus* erlotinib alone in nonsquamous NSCLC harboring mutant EGFR [76]. The ORR was 69% in the combination group *versus* 64% in the erlotinib group. However, the disease control rate is higher for erlotinib plus bevacizumab than erlotinib (99% *versus* 88%). Specifically, there was an improvement of PFS by approximately 6 months when bevacizumab was added to erlotinib (16.0 *versus* 9.7 months). Combination treatment did not produce new safety issues. The JO25567 study was the first to obtain clinically meaningful data that confirmed the efficacy of combined bevacizumab and EGFR-TKI in the first-line management of advanced NSCLC with EGFR-activating mutation. To date, the OS data are premature, and a larger phase III trial will be required to establish the efficacy of this combination therapy. The results from BELIEF trial showed that bevacizumab plus erlotinib benefited patients with sensitive EGFR mutations, with an increased ORR of 76.1% and PFS of 13.8 months. Even a subgroup of patients with a known EGFR T790M mutation had an improved PFS (16.0 months) and ORR (70.3%) [77]. Moreover, a small phase II trial evaluated the efficacy of gefitinib in combination with bevacizumab in treatment for EGFR-mutant metastatic NSCLC. The ORR was 73.8%, and 2 patients had a complete response to treatment; the median PFS in all patients were 14.4 months *versus* 18.0 months in a subgroup of patients with exon 19 deletions [78]. In May 2016, the EMA approved the use of bevacizumab plus an EGFR TKI as a first-line therapy for unresectable, metastatic and recurrent NSCLC.

Other researchers have attempted to evaluate the efficacy of antiangiogenic agents in combination with EGFR TKIs as maintenance or second-line therapy. The early ATLAS trial did not confirm that a two-drug maintenance regimen consisting of bevacizumab plus erlotinib can improve OS for advanced NSCLC patients who received four cycles of induction chemotherapy plus bevacizumab, although a benefit in PFS was recorded, and this combination regimen was generally tolerated well [79]. In a previously reported phase II trial, combining bevacizumab with erlotinib did not prolong PFS compared to docetaxel or pemetrexed monotherapy alone for patients who were refractory to first-line chemotherapy, although the one-year OS rate was numerally higher than that observed in the bevacizumab-erlotinib arm (57.4% compared with 33.1% for chemotherapy alone) [37]. An impossible explanation would be that most enrolled patients were EGFR mutation-negative in all study arms. Moreover, the BeTa trial demonstrated that bevacizumab in combination with erlotinib failed to produce a survival benefit for NSCLC patients in the second-line setting, irrespective of the EGFR mutation status [80]. Second-line bevacizumab plus EGFR TKIs were also evaluated in selective patients with NSCLC who harbored mutant EGFR. A retrospective study showed that the T790M mutation could be inversely associated with the efficacy of EGFR TKI rechallenge plus bevacizumab in a subgroup of EGFR-mutant patients [81]. Overall, bevacizumab in combination with EGFR TKIs might be a well-tolerated treatment strategy for patients with EGFR mutations, even for a subset of patients with primary resistance to gefitinib or erlotinib.

Several trials evaluating this combination strategy are ongoing, such as the BEVERLY trial to evaluate the efficacy of erlotinib plus bevacizumab or erlotinib in advanced NSCLC with sensitive EGFR mutations in the first-line setting. This trial will confirm the results from previous phase II study, and this regimen is expected to become the standard care for this population [82]. Future developments may also focus on the combination of antiangiogenic agents with third-generation TKIs, for the management of EGFR-mutant NSCLC with or without brain metastases (NCT02803203 and NCT02971501). Furthermore, a phase Ib, dose-escalation trial was designed to investigate the safety and pharmacodynamics of crizotinib plus an individual VEGF inhibitor (axitinib, sunitinib, bevacizumab or sorafenib) in advanced solid tumors, despite the success of antiangiogenic therapy in multiple treatment settings (NCT01441388). However, this trial did not enroll patients and has been withdrawn.

### Ramucirumab plus EGFR TKIs

The RELAY trial is an ongoing phase Ib/III trial that assesses the efficacy and safety of first-line ramucirumab in combination with erlotinib in patients with advanced NSCLC who harbor a sensitive EGFR mutation (NCT02411448) [83]. The phase Ib part of this trial will evaluate the dose-limiting toxicity during 4 weeks of therapy. The phase III part will compare first-line ramucirumab-erlotinib to placebo-erlotinib. The primary endpoint is PFS based on investigator assessment, and patients will be excluded if they harbor a known EGFR T790M alteration. This study will reveal whether the addition of ramucirumab to erlotinib further improves the efficacy of first-line erlotinib, which is a standard care for advanced NSCLC whose tumors have EGFR mutations.

### VEGFR-TKIs plus EGFR TKIs

Another phase II trial demonstrated that adding sorafinib to EGFR-TKIs did not significantly enhance PFS (3.38 months with combination therapy, and 1.94 months with erlotinib alone) and OS (7.62 months with combination therapy, and 7.23 months with erlotinib alone) in unselected advanced NSCLC who progressed following first-line chemotherapy [84]. Interestingly, the combination of erlotinib plus sorafenib provided a survival benefit in a subset analyses to patients harboring wild-type EGFR or FISH-negative EGFR had a PFS and OS compared with single-agent erlotinib. Sunitinib has also been investigated in combination with erlotinib for NSCLC that failed first-line chemotherapy. Specifically, a randomized, multicenter trial by Groen et al. showed that sunitinib plus erlotinib did not produce a PFS benefit in the second-line setting (2.8 *versus* 2.0 months) [85], whereas a subsequent larger phase III study demonstrated that sunitinib plus erlotinib was superior to erlotinib alone, with a significant improvement of PFS (3.6 months *versus* 2.0 months) [86]. The median OS was not different in the two groups (9.0 months in the combination group *versus* 8.5 months in the erlotinib group). Additionally, grade 3 or 4 drug-related toxicities were more frequent with combination therapy.

### Rh-endostain plus EGFR TKIs

Recently, some investigators have assessed the efficacy of combined rh-endostain plus EGFR TKIs in metastatic EGFR-mutant NSCLC [87]. For example, Zhao et al. reported a retrospective study including 10 patients with an exon 19 del or exon 21 L858R mutations. These patients received the first-line combination of rh-endostain plus icotinib. The preliminary results demonstrated a clinical ORR of 60% at 24 weeks and a mean decrease in tumor size of 32.5%. The toxicity profile was consistent with that reported in previous clinical trials of rh-endostain or icotinib.

## COMBINATION OF ANTIANGIOGENIC AGENTS WITH IMMUNE CHECKPOINT INHIBITORS

**Figure 1:**
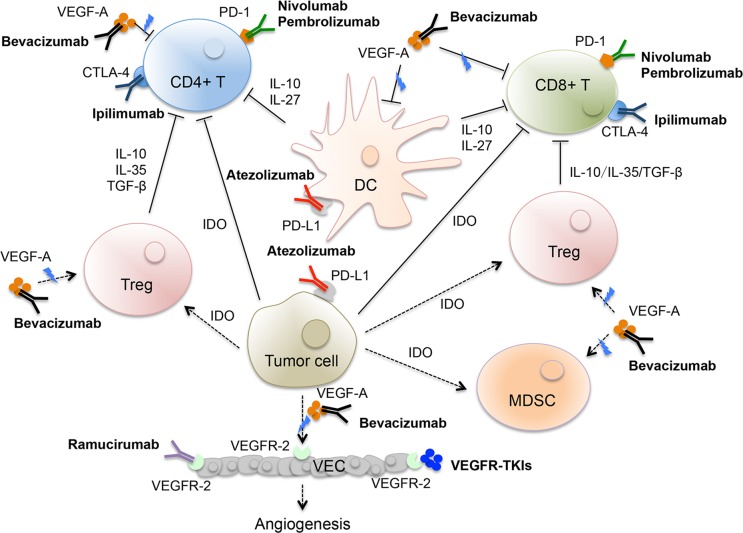
VEGF-A-mediated inhibition of immune response and potential combination strategy with angiogenesis inhibitors and immunotherapy Immune checkpoint inhibitors anti-PD-1 antibidy (nivolumab and pembrolizumab) or anti-PD-L1 antibody (atezolizumab) can combine with antiangiogenic agents (bevacizumab, ramucirumab, and oral small-molecule EGFR-TKIs) for targeting tumor. VEGF-A, vascular endothelial growth factor A; PD-1, programmed cell death protein-1; PD-L1, PD-ligand 1; CTLA-4: cytotoxic T-lymphocyte-associated protein 4; TGF-β, transforming growth factor-β; IL-10, interleukin-10; IL-35, interleukin-35; IL-27, interleukin-27; Treg, T- regulatory cell; DC: dendritic cell; VEC: vascular endothelial cell; CD4+ T: cluster of differentiation 4+ T cell; CD8+ T: cluster of differentiation 8+ T cell; IDO, indoleamine 2, 3 -dioxygenase; MDSC, myeloid-derived suppressor cell; VEGFR-2: vascular endothelial growth factor receptor-2; VEGFR-TKIs: vascular endothelial growth factor receptor tyrosine kinase inhibitors.

Blocking either of programmed cell death protein 1 (PD-1) or programmed cell death protein ligand 1 (PD-L1) using specific antibodies has been developed as a successful therapeutic strategy for advanced disease. Specifically, nivolumab [88, 89], pembrolizumab [90], and atezolizumab with docetaxel [91] have been approved for treating metastatic NSCLC in the second-line setting. Furthermore, pembrolizumab demonstrated a clinical benefit as a monotherapy for PD-L1-positive NSCLC in the first-line setting [92]. Recently, studies suggest that blocking angiogenesis may increase the efficacy of immune checkpoint inhibitors, and the combination of these two approaches is generally tolerable (Figure 1). Moreover, comprehensive preclinical and clinical rationale data sustain the hypothesis that anti-VEGF could synergize with immunotherapy to benefit the patients [93, 94]. First, the pro-angiogenic factor VEGF-A is immunosuppressive and helps tumor cells evade immune surveillance by inhibiting T-cell infiltration and trafficking into the tumor and preventing the maturation of dendritic cells [95]. Furthermore, antiangiogenic agents stimulate the immune response by increasing the infiltration of CD4+ T and CD8+ cells into tumors [96]. Second, antiangiogenic agents also exerts a regulatory role in the inhibition of immune signals, including the inhibition of T-regulatory cell proliferation, myeloid-derived suppressor cell function, dendritic cell maturation, and PD-1 expression in tumor-infiltrating T cells [97].

### Bevacizumab plus immune checkpoint inhibitors

A phase I trial evaluated the efficacy and safety of switching to nivolumab maintenance therapy as a monotherapy or combined with bevacizumab for patients with metastatic NSCLC after completing 4 cycles of the first-line platinum-containing doublet chemotherapy (NCT01454102; CheckMate 012) [98]. A total of 12 NSCLC patients with nonsquamous histology received nivolumab and bevacizumab maintenance treatment, and 13 with nonsquamous histology and 8 with squamous histology received nivolumab monotherapy maintenance treatment. The median PFS was 37.1 weeks for nivolumab plus bevacizumab. In the nivolumab monotherapy group, the median PFS for squamous and nonsquamous patients was 16 and 21.4 weeks, respectively. The ORR in the nivolumab plus bevacizumab group was 8% and that for the nivolumab monotherapy group was 10%. The 1-year OS rate was 75% for patients with nivolumab plus bevacizumab treatment. Four patients experienced grade 3 adverse effects, and treatment-related grade 4 adverse effects were also observed in the nivolumab plus bevacizumab group. The results of this trial suggest that switching to nivolumab combined with bevacizumab maintenance therapy results in a PFS similar to that seen with other agents used in a maintenance setting following platinum-containing chemotherapy for patients with metastases. Moreover, the side-effect profile was acceptable. Several ongoing trials are designed to assess the efficacy of bevacizumab in combination with pembrolizumab (NCT02681549) or atezolizumab (NCT02366143) for advanced NSCLC (Table 4).

Furthermore, several investigators have attempted to evaluate the safety and efficacy of combining bevacizumab and checkpoint inhibitor immunotherapies in the management of other types of human cancer. For example, a phase I trial was conducted in advanced melanoma and demonstrated that combined bevacizumab and the CTLA-4 checkpoint inhibitor ipilimumab produced promising results. Forty-six patients received different combinations of ipilimumab and 15 mg/kg or 7.5 mg/kg bevacizumab. Eight patients exhibited PR, and 22 patients experienced stable disease, with a median OS of 21.5 months. Further analyses showed extensive CD8+ and macrophage cell infiltration within tumors, with an increasing number of circulating T cells and antigalectin antibodies [99]. The efficacy of bevacizumab plus atezolizumab was also investigated in advanced renal cell carcinoma. Combination treatment with bevacizumab and atezolizumab produced a promising ORR of 40%. The best response in another 5 patients was stable disease (NCT01633970) [100]. In comparison, the ORR for atezolizumab monotherapy in a previous phase I trial was 15% [101], whereas this rate was 10% for bevacizumab monotherapy at a high-dose in this setting [102]. Grade 3 or 4 adverse effects (6/10) due to atezolizumab therapy were not observed. In a dose-escalation study, 15 mg/kg or 10 mg/kg bevacizumab and a fixed dose of 200 mg atezolizumab treatment did not produce dose-limiting toxicity or serious adverse events, suggesting that this combination is safe and recommended for a subsequent ongoing phase II study (NCT02348008). In a multicenter phase Ib trial (NCT01633970), the clinical efficacy of bevacizumab plus atezolizumabin was evaluated for refractory advanced colorectal cancer, and the efficacy of bevacizumab plus atezolizumab plus FOLFOX chemotherapy was evaluated in oxaliplatin-naïve patients [103]. The unconfirmed ORR was 8% (1/13) in patients treated with bevacizumab plus atezolizumab and 36% (9/25) in patients with oxaliplatin-naïve patients treated with bevacizumab plus atezolizumab plus chemotherapy. Moreover, the unconfirmed ORR was 44% (8/18) for patients treated with first-line bevacizumab plus atezolizumab plus chemotherapy. Patients treated with bevacizumab plus atezolizumab with or without chemotherapy tolerated treatment well in both arms, with no unexpected toxicities.

### Ramucirumab plus immune checkpoint inhibitors

In a phase I trial including previously treated advanced NSCLC, gastric or gastroesophageal junction adenocarcinoma or urothelial carcinoma received combined treatment with ramucirumab and pembrolizumab. Preliminary safety results from the dose-limiting toxicity portion of the trial did not identify unexpected safety concerns, and dose-limiting toxicity was not observed in patients with NSCLC (NCT02443324) [104]. This study is the first to assess the synergistic effect of ramucirumab, a VEGFR-2 antibody, and pembrolizumab, a PD-1 antibody, to simultaneously target both angiogenesis and immunosuppression. Recently, Herbst et al. presented the interim data of the clinical trial at the 2016 ESMO Congress. Specifically, the disease control rate reached 85%, and 8 patients exhibited an objective response and reduction in tumor size, with a median time to response of 1.45 months. Moreover, the evaluation of objective responses is ongoing in all patients responsive to the combination treatment (Study Of Ramucirumab Plus Pembrolizumab Shows Promise In NSCLC ESMO Abstract 2428).

### VEGFR-TKIs plus immune checkpoint inhibitors

Numerous trials have attempted to explore the efficacy of VEGFR-TKIs in the treatment of metastatic NSCLC, but the majority of these treatments failed to prolong PFS and OS and were associated with significantly increased toxicity. Based on data from the LUME-lung 1 trial, nintedanib is the only antiangiogenic drug that has been approved by the EMA in combination with docetaxel as a second-line treatment for metastatic NSCLC with adenocarcinoma histology after first-line chemotherapy failure. However, data from clinical trials assessing the safety and efficacy of combined treatment with antiangiogenic TKIs and immune checkpoint inhibitors in NSCLC are not available. Nevertheless, a trial assessing the combination of nintedanib and pembrolizumab in metastatic NSCLC is currently ongoing (NCT02856425). Recently, a phase I study (CheckMate 016) was designed to determine the effects of the combination of the antiangiogenic TKIs pazopanib or sunitinib and nivolumab on metastatic renal cell carcinoma that had received more than 1 prior systematic treatment [105]. The ORR was 52% in the sunitinib plus nivolumab group and 45% in the pazopanib plus nivolumab group. The PFS rates at 24 weeks were 78% and 55% for the two arms. Although most patients experienced grade 3 to 4 toxicities, these events were relatively common and manageable (NCT01472081).

## ANTIANGIOGENIC AGENT MONOTHERAPY

Antiangiogenic agent monotherapy exhibits a lower response rate than combination therapy with an antiangiogenic agent and another therapeutic strategy. For example, a phase I trial including 37 patients diagnosed with different solid tumors showed that patients received ramucirumab at a dose of 2 to 16 mg/kg once weekly [106], but only 4 patients exhibited a confirmed PR. However, lung cancer patients were not included in this study.

Moreover, many studies have attempted to evaluate the benefit of antiangiogenic TKIs to advanced NSCLC patients who progressed on second-line or subsequent lines of therapy. In the recent MISSIN trial, NSCLC patients were randomized to receive sorafenib (*n* = 350) or placebo (*n* = 353) as a third-line therapy [107]. The OS was designed as primary endpoint. Monotherapy treatment with sorafenib failed to improve the OS (8.2 *versus* 8.3 months; *p* = 0.47), despite significant improvement in PFS (2.8 *versus* 1.4 months; *p* < 0.0001). A total of 17 patients (4.9%) in the sorafenib arm and 3 patients (0.9%) in the placebo arm achieved a PR. Interestingly, patients harboring an EGFR mutation (*n* = 89) exhibited improvements in OS (13.9 *versus* 6.5 months; *p* = 0.002) and PFS (2.7 *versus* 1.4 months; *p* < 0.001) in response to sorafenib monotherapy (8.2 *versus* 8.3 months; *p* = 0.47). Similarly, another oral inhibitor targeting VEGFR, EGFR and RET signaling, vandetanib, failed to improve OS *versus* placebo following prior therapy with an EGFR TKI and one or two chemotherapy regimens [108]. A recently published Chinese trial of sorafenib in advanced NSCLC who progressed on EGFR TKI indicated that sorafenib did not improve survival (PFS: 3.7months; OS: 7.4 months). The disease control rate was 32.8% [109]. Additionally, other antiangiogenic agents only showed modest trends in survival benefits but increased toxicity that reflected known antiangiogenic effects [110–114]. Given a lack of survival improvement and an increasing risk of death in these trials, oral multikinase inhibitors of angiogenesis have not been incorporated into treatment algorithms for advanced NSCLC as a monotherapy.

Recently, a phase II trial assessing the ability of the anti-VEGFR-2 TKI apatinib to improve PFS *versus* placebo in metastatic nonsquamous NSCLC after the failure of more than two lines of treatment [115]. Specifically, a total of 135 Chinese patients received apatinib monotherapy (*n* = 90) or placebo (*n* = 45). The median PFS significantly differed between the apatinib and placebo groups (4.7 *versus* 1.9 months), and increases in the ORR and DCR were also observed in patients who received apatinib (12.2% and 68.9%) *versus* patients who received placebo (0% and 24.4%). The AEs reported in the apatinib arm were manageable. In the subsequent phase III trial, the investigators will further assess the efficacy and safety of apatinib in as a third- or fourth-line treatment for metastatic nonsquamous NSCLC (NCT01287962). However, most patients who receive third- or fourth-line therapy respond worse than patients receiving the first- or second-line treatment, and treatment will more significantly improve their quality of life. Anti-VEGFR TKI monotherapy as a third or subsequent line of therapy remains of questionable benefit and should not be considered as part of the current standard of care.

## CONCLUSION

The suppression of tumor-induced angiogenesis has identified as an attractive treatment strategy for advanced NSCLC as well as other types of cancer. However, antiangiogenic agents alone exhibit limited clinical efficacy but may be considered as a choice in the third-line setting. Bevacizumab has been approved as an antiangiogenic monoclonal antibody in the first-line treatment of advanced NSCLC, whereas second-line us with ramucirumab showed a survival benefit. To date, bevacizumab in combination with chemotherapy is recommended in nonsquamous NSCLC who are free brain metastases, major bleeding or thrombotic disorders. In contrast to bevacizumab, ramucirumab plus chemotherapy can be used in the second-line setting without histological limitation. Moreover, most antiangiogenic TKIs fail to reach the primary endpoint or exhibit meaningful improvements in survival, with the exception of nintedanib. Specifically, it produced a survival benefit in the LUME Lung-1 trial when added to docetaxel as a second-line therapy. Nevertheless, some challenges remain to be overcome, including the lack of predictive biomarkers to help select patients who would benefit from antiangiogenic therapy and developing more potent antiangiogenic agents beyond the currently approved agents bevacizumab and ramucirumab. It is also interesting to investigate whether antiangiogenic agents should be used constantly instead of periodic treatment with chemotherapy. Although ‘vascular normalization’ was an alternative strategy to partially reduce tumor vessel number and induce maturation of vessels during antiangiogenic therapy, this temporary treatment window for drug delivery is still difficult to predict.

In addition to the combination of antiangiogenic agents with chemotherapy, antiangiogenic agents in combination with TKIs also produced promising results. For example, bevacizumab plus erlotinib significantly improved PFS in the BELIEVE and JCO25567 studies. Other similar studies, including the ACCRU (NCT01532089, bevacizumab plus erlotinib) and RELAY study (NCT02411448, ramucirumab plus erlotinib), are ongoing. These trials are expected to show a meaningful improvement in OS for advanced NSCLC treated with dual targeted drugs. Furthermore, given the great success of immunotherapy with immune checkpoint inhibitors in NSCLC and the immunosuppressive potential of angiogenic factors, antiangiogenic agents and immunotherapy may exhibit potentially synergistic anticancer activity. Compared with the combination of antiangiogenic therapy and chemotherapy, the combination of antiangiogenic therapy with immunotherapy is expected to have an acceptable toxicity profile. Currently, available data on such combinations are preliminary and immature, whereas combined antiangiogenic therapy and immunotherapy with checkpoint blockade is a promising strategy for the future clinical management of metastatic NSCLC. Theoretically, the combined inhibition of two distinct but related pathways, such as the VEGFR and the EGFR or the PD-1/PD-L1, could produce a more sustained suppression of cancer-related angiogenesis and tumor growth. However, the current use of antiangiogenic agents does not depend on the selection of particular molecular characteristics in clinical practice, and the correlation of PD-L1 expression and other immune predictors with clinical response of immunotherapy in advanced NSCLC has not been verified, except for pembrolizumab [90]. Thus, the combination of antiangiogenic and immunotherapy strategies is expected to be associated with many challenges and may complicate the clinical prediction and evaluation of targeting angiogenesis and immunotherapy concurrently. Overall, to date, antiangiogenic treatment should be considered as a part of combination and personalized therapy including chemotherapy, small-molecule TKIs and immunotherapy, especially in the first-line treatment of metastatic NSCLC [116–117].
